# Digital twins as education support in construction: a first development framework based on the Reference Construction Site Aachen West

**DOI:** 10.1007/s41693-022-00070-7

**Published:** 2022-04-06

**Authors:** Rushi Dai, Sigrid Brell-Çokcan

**Affiliations:** Individualized Production in Architecture, RWTH Aachen University, Aachen, Germany

**Keywords:** Digital twin, Unreal Engine, Construction site, Education, Real-time interaction, MQTT, Automation and digitization

## Abstract

The Reference Construction Site Aachen West, located in Aachen, Germany, is a campus-based living lab for research and university education in construction. To ensure and improve the education quality, this paper researches a new study tool in the form of a digital twin framework powered by the Unreal Engine. By implementing MQTT, an IoT communication protocol, the digital twin realizes synchronicity of cyber-physical bi-directional data flows. Representing the virtual Reference Construction Site, an online multiplayer application was developed as a use case to answer the research questions: (1) how to design the digital twin and structure in an extendable and accessible development framework for different users and (2) how to support education in construction with this digital twin. To validate the concept, a group of students were invited online to explore the application and provide user feedback. Results show positive effects of the digital twin on enhancing the quality of the online education in construction with its real-time interaction and data from on-site machinery and processes. The digital twin is planned to be transferred to research and construction projects.

## Introduction

Benefiting from the development of digital technologies, the construction industry is currently learning from Industry 4.0 and undergoing an unprecedented digital transformation. To accelerate the speed of innovation, the construction industry not only integrated cutting-edge technologies and methodologies, but also organized interdisciplinary education to foster talents the new direction of automation and digitization. However, the current upgrade is at an early stage. The outbreak of COVID-19 had a huge impact on this industry. Survey results from the 2021 CHAS report showed, that almost 70% of respondents have ceased projects during the pandemic and 80% have cancelled or postponed some of their projects (CHAS 2021). A similarly strong impact could be observed on education in construction as well. Because a large number of the education activities events are dependent on on-site machinery and processes, the pandemic prevention measures, such as social distancing and working from home regulations, largely limited physical access to facilities. Additionally, many international students failed to obtain visas to study abroad. As a result, most teaching activities and practical courses were obliged to be conducted digitally and remotely, which added difficulties in both teaching and learning.

To better improve education quality and promote innovation in the construction industry, this paper demonstrates a game-engine-enabled digital twin (DT) and its development framework supporting education in construction. Powered by Unreal Engine (UE), the interactive DT provides a new study tool with real-time data connecting to construction sites to assist the online education. As a use case, the Reference Construction Site Aachen West -a campus-based living lab for research and university education in Aachen, Germany- was chosen as an application scenario for the DT development. This paper addresses the following research questions:

- How is the DT designed and its development framework structured to be extendable and accessible for users from different disciplines?
- How can a DT, powered by UE, support and enhance the education in construction?

## Related work

### Digital twin

The concept of DT was introduced by Michael Grieves at University of Michigan in 2003 for Product Lifecycle Management (M G 2015) and the first practical definition of DT was originated by NASA to simulate and visualize undergoing changes of a spacecraft in an extremely hostile environment (Negri et al. 2017). A DT is a dynamic virtual representation of a physical object or system, using real-time data for bi-directional communication, interaction and simulation (Brilakis et al. 2019). With its characteristics of connectivity, visualization, programmability and modularity, different DTs have been successfully applied in the industries of manufacturing, automotive, and urban planning. By connecting to a real manufacturing factory, DT has helped in integrating dynamic and complex production processes into an easy-to-use and intuitive cooperation platform between diverse experts (Yildiz et al. 2021).

As construction is well-known for being dynamic, complex and multi-disciplinary, the concept of DT also attracted the attention of the construction industry. Opoku et al. researched 22 papers of DT in the construction industry from 2010 to 2020. The research found that the DT application had been slow until the year of 2019 (Opoku et al. 2021). Since then, more applications emerged centered on BIM and IoT technologies. The combination of BIM and wireless sensor networks created an active model to assist decision making, detect anomalies, estimate time and cost, as well as provide a cooperation platform (Lin and Cheung 2020). However, there are differences between BIM and DT, such as, BIM works with static rather than real-time data (Khajavi et al. 2019). The word ’real time’ here refers to hard real time system (e.g., a DT system), in which a single failure to meet the deadline may lead to complete system failure. While a soft real time system (e.g., simulation system in BIM) is a system in which failures to meet the deadline are considered as performance degrading (Mandula 2018). Currently, some leading industries have developed cloud-based DT for AEC industry, such as Autodesk Tandem and Bentley iTwin. Based on their own mature product ecology, these products are already powerful in data integration, software API and online-collaboration. Nevertheless, users must pay high fees for industrial service, and the freedom of development is relatively low.

Some studies have seen potentials of DT in the engineering education. Results from a research proved that, the real-time interactive DT has succeeded in increasing the motivation of students and improving the learning quality in the study of machine automation (Liljaniemi 2020). Another research led by the Faculty of Built Environment at University of New South Wales Sydney showed the excellent education support of DT in Construction Management and Engineering. Students have gained similar on-site experience and cognition in the simulated environment during the COVID-19 (Sepasgozar 2020).

### Unreal Engine for digital twin

A game engine, such as Unity and UE, is the underlying software of a real-time interactive game, providing a platform, in which code, data and art assets are produced by different experts to make a game. This complex system of intertwined layers relating to various hardware and software generally consists of an Engine Core and a larger number of components, including graphics rendering, collision detection, physics simulation, networking and so on Toftedahl and Engström (2019). Because of the powerful features in game engines, other industries are starting to use these tools for DT development. In the field of manufacturing, examples of using Unity for DT in production management and machine automation are frequent (Yildiz et al. 2021; Ke et al. 2019). Since the powerful 3D rendering in UE, smart city, a technologically modern concept for city digitization, has set its sights on this engine. According to the official website of UE, the company 51World have succeeded in creating a DT of Shanghai with UE for autonomous decision-making. Meanwhile, the Buildmedia in New Zealand was using UE to develop a DT for city’s transport capacity based on a GIS-accurate model of Wellington (Weir-McCall 2020).

After comparison of different game engines, UE was chosen in this paper to be the development tool because of its high visual fidelity, vast developer toolkits, accessibility, fast network synchronization and cross-platform deployment (Christopoulou and Xinogalos 2017). Coding in C++ and Blueprint Visual Programming, UE enables both programmers and non-programmers to work on the same platform. Moreover, many plug-ins in UE, such as Datasmith, support users from AEC industries with a seam-less workflow between UE and other 3D software, including Rhino, Sketchup, SolidWorks and Revit (ifcModel) (Documentation 2021).

### MQTT

To realize the interaction between the physical and digital world, IoT (Internet of Things) provides access to real objects and connection to virtual replicas in digital twins (Immerman 2020). Regrading data and connectivity, IoT is one of the key enablers and the backbone of digital twin. It builds up a foundation of communication via IoT nodes (sensors/actuators) and network technologies, so that information can flow between different systems and things which through connectivity become smart.

Message Queuing Telemetry Transport (MQTT) is a standard messaging protocol for IoT, which allows different systems/devices to communicate remotely with one language (MQTT 2020). MQTT is widely applied in industries, such as manufacturing, construction and automotive, due to its benefits of light weight and reliable message transportation. MQTT transfers messages through a central broker over TCP using Publish-Subscribe structure. With a small code footprint and minimal bandwidth usage, it can be easily implemented in third party software and hardware via a wide variety of programming languages, such as C#, Python and C++.

In this paper, MQTT was chosen as the main IoT protocol for the bi-directional communication, because it is a light-weight method and has already been applied as a communication protocol in our facilities and construction sites. The use case developed in this paper demonstrates the potentials of the DT development framework to embed IoT technologies.

## Methodologies

During the DT development, the object-oriented method learned from Information Engineering was applied (Graba 1998). An object, which contains attributes (data) and working functions (methods), can be defined as a digital representation of a real-world physical object. The fundamental concepts of the object-oriented method are (1) encapsulation—wrapping up associated data and methods, (2) data abstraction—instantiating type class into individual objects, (3) inheritance—organizing classes into hierarchies, and (4) polymorphism—sharing characteristics and interfacing between objects. According to specific use cases and target users in this paper, different types of objects were categorized in the hierarchy of the development framework, such as the objects of construction machinery, building elements, communication methods and so on. Powered by UE, this framework is designed for organizing a complex system in a structured and reusable way.

For concept validation, a DT use case for education was tested by a group of 15 students from an international master programme Construction&Robotics. These students were invited to experience a virtual tour in the DT to provide user feedback. The user feedback was considered to determine the future directions of the DT development.

### Design of the digital twin and its development framework

**Fig. 1:**
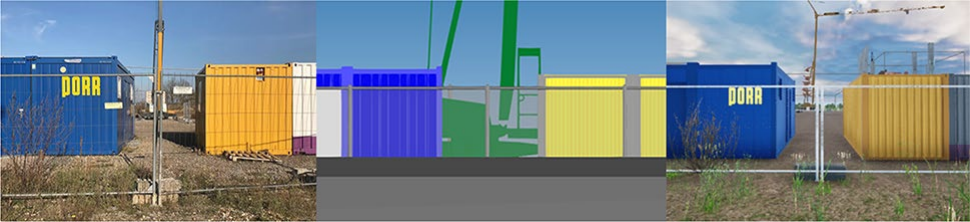
From left to right: (1) Real scene at the Reference Construction Site (2) IfcModel in IFC Viewer (3) IfcModel-based DT in UE

The DT in this paper replicates the Reference Construction Aachen West in a virtual world. The Reference Construction Site was previously constructed based on an ifcModel. Via Datasmith, the ifc file was imported and converted into UE-format assets to build up the 3D environment in UE. By changing materials and adding environmental assets, photorealistic models were generated (Fig. 1). This section presents a development framework to program these 3D models into interactive and communicative twins.

**Fig. 2:**
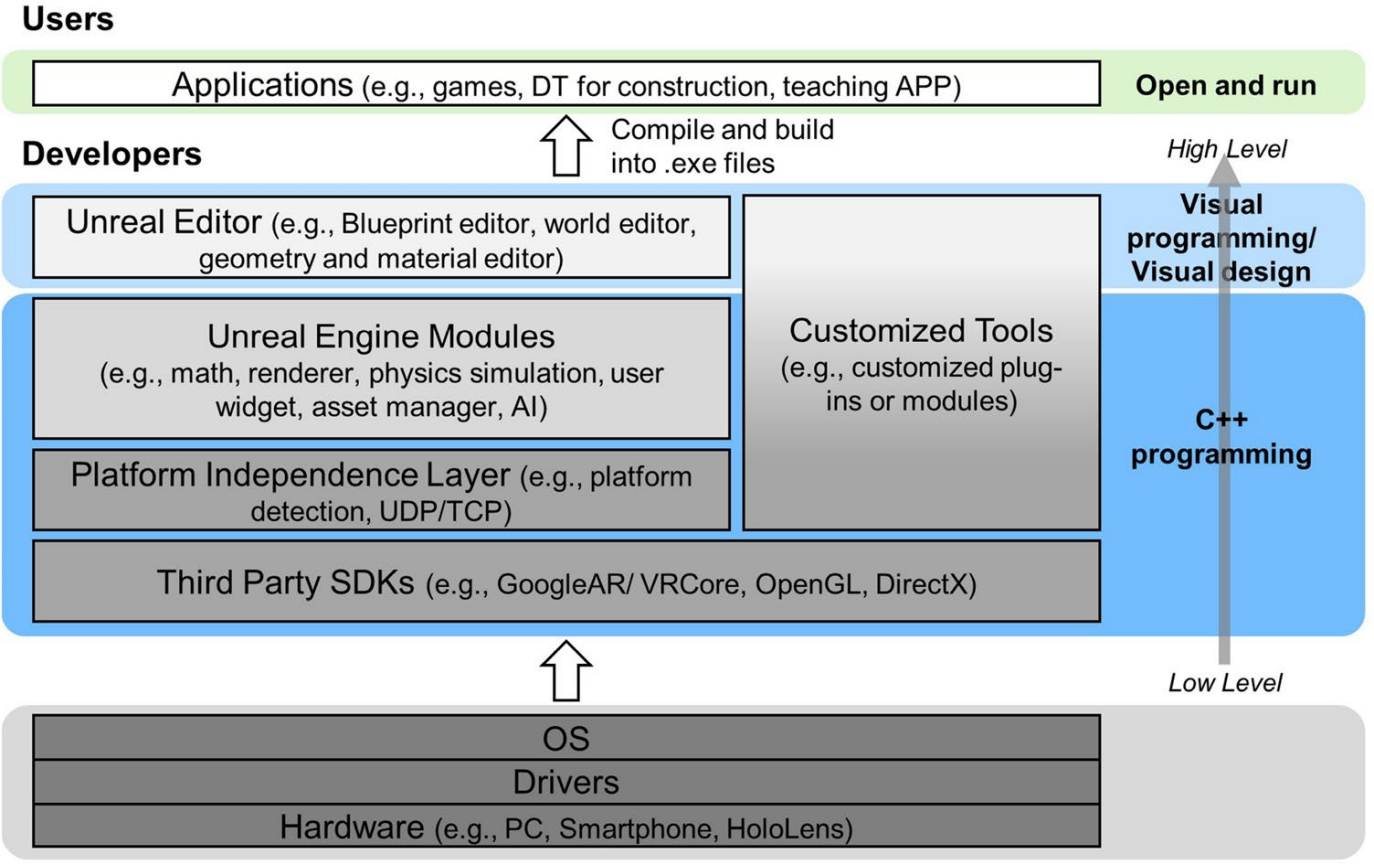
Tool architecture of Unreal Engine (Gregory 2009)

As shown in Fig. 2, the UE platform consists of layers from low level to high level: (1) Third Party SDKs to interface different hardware and software, (2) Platform Dependency Layer for the engine to run on different hardware platforms, (3) Customized Tools written by individuals, e.g., the MQTT modules in this paper, (4) UE Modules for core functions, e.g., graphic rendering, math and physics simulation, and (5) UE Editors, e.g., world editor and Blueprint visual programming. UE manages the underlying architecture and provides a collaborative platform for programmers, designers and engineers. This builds up the foundation of the development framework for the construction DT.

**Fig. 3:**
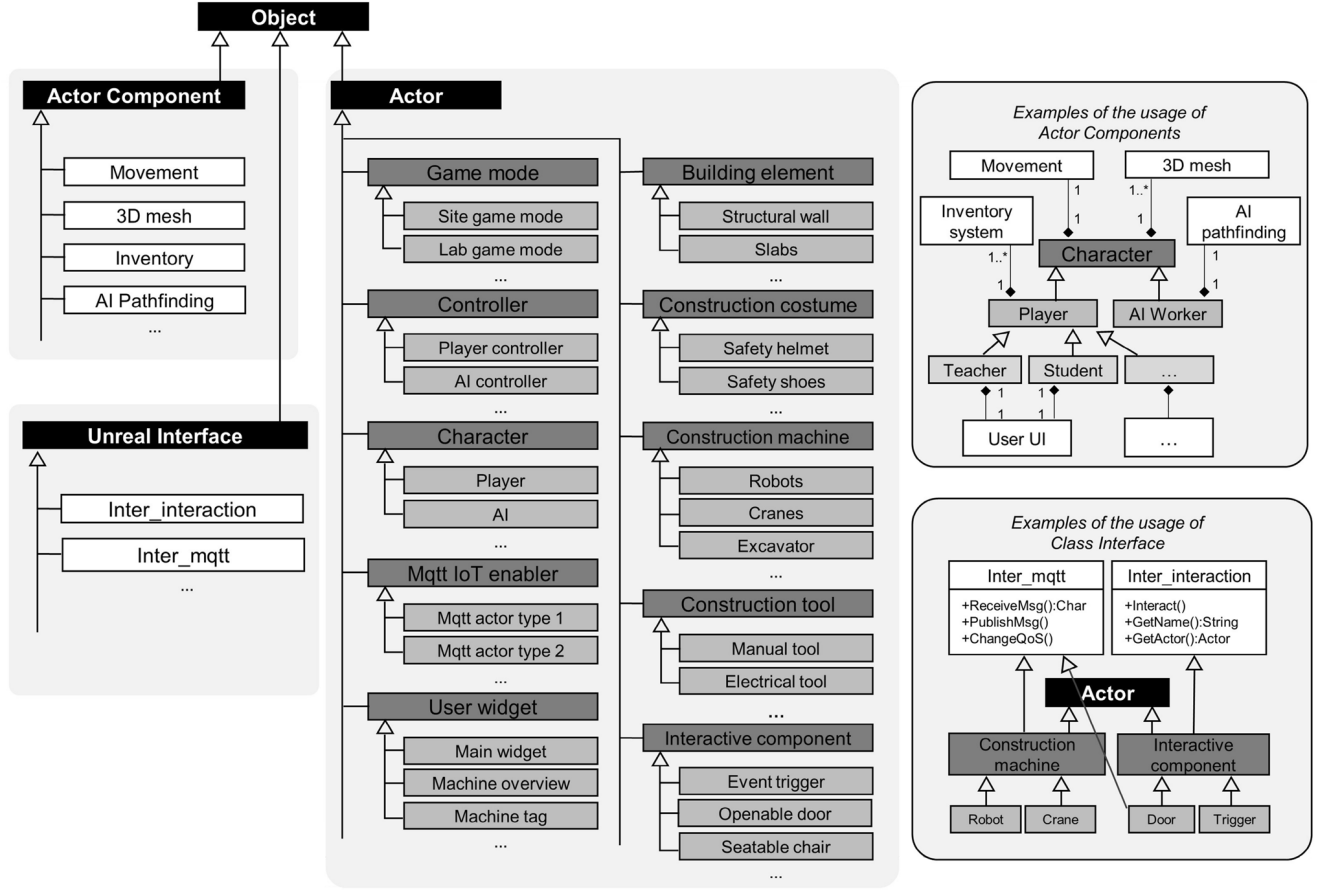
The UML diagrams of class hierarchy in the DT development framework

The development framework can be embodied as a UE project package with construction-related templates and modules. The UE project package contained construction relevant assets, C++ parent classes (for machinery, tools, building elements, user interfaces and more) and their Blueprint child actors for visual-programming the shader, interaction, gameplay logic and so on. Each parent class owns standardized attributes, operation methods, associated components and common features shared among other classes. Their child actors can be directly dragged and dropped into the Unreal World Editor for run-and-play simulation. Figure 3 demonstrates the UML diagrams of the class hierarchy structured in the DT framework. As a result, developers can directly construct 3D environment and application logic based on the class templates and pre-defined actors without starting from scratch. Besides, it is also easy to reproduce similar DT projects by simple migrations.

**Fig. 4:**
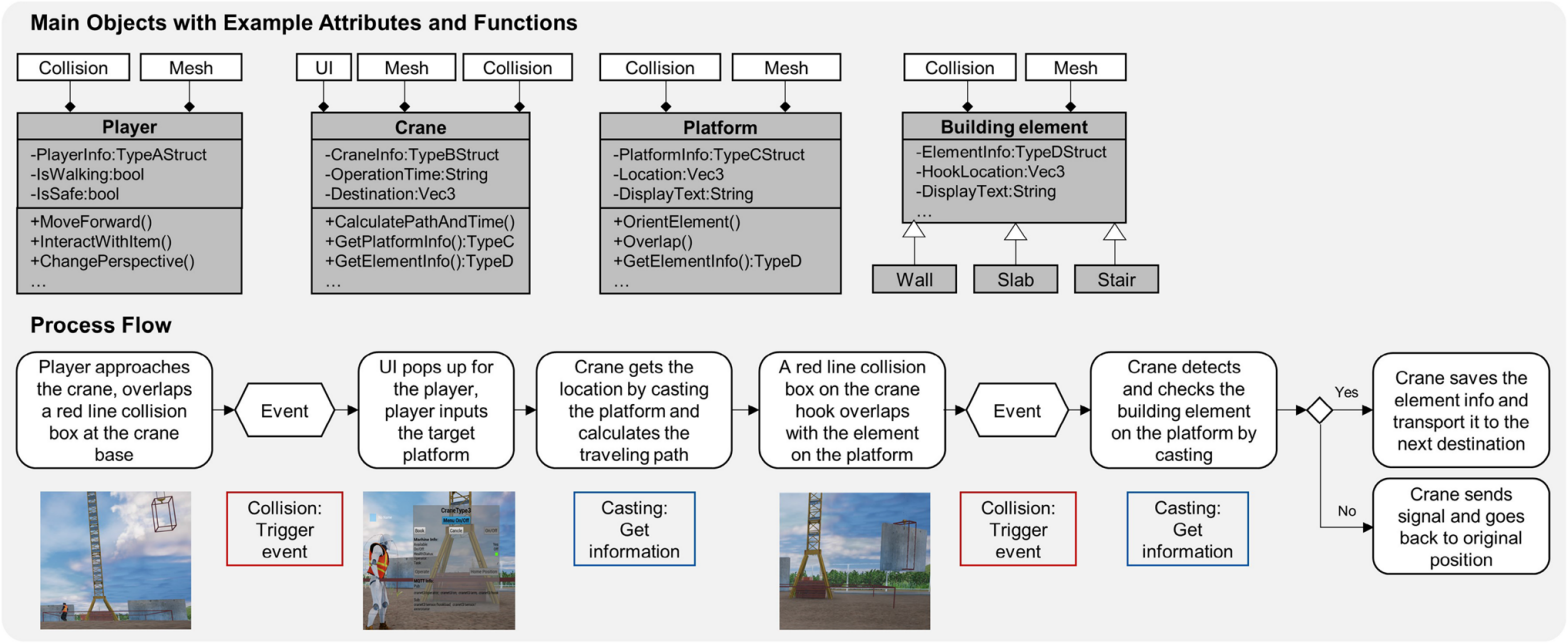
Event-driven process, an example of transporting a building element by a crane

As the construction processes consist of many objects that work in sequences with bidirectional information flow, the DT utilizes three communication methods from UE to register and trigger events in real time: (1) collision, (2) casting, and (3) interfaces. Figure 4 shows a process diagram of a crane transportation. It is about how to use collision boxes to trigger events by overlapping with objects and then cast the objects into target classes for validation check and data acquisition (e.g., object name and operation time). Interfaces were applied as common features between different classes to trigger similar events. Examples can be seen in the MQTT communication between virtual and physical objects. Inheriting from the MQTT interface class, the different types of MQTT-enabled objects can receive messages from the designated broker and trigger events of operation, such as I/O, changing operation speed, notification and more (Fig. 3).

### Use case supporting education

**Fig. 5:**
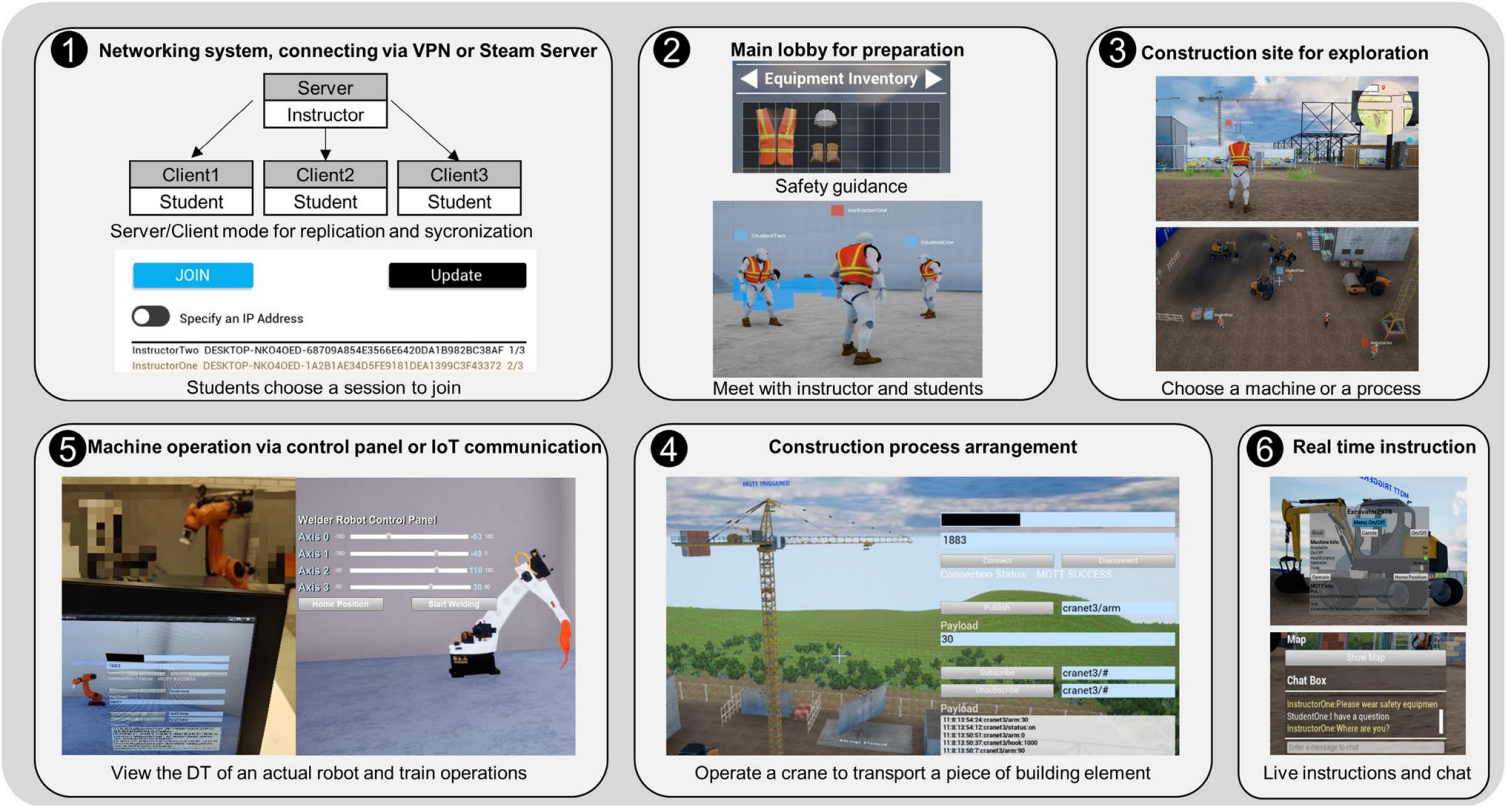
The six core functions of the multiplayer online DT

One of the applications derived from the template of the development framework is a multiplayer online DT. Locating in one LAN, a user can host a server for other users to join in the same network session. In this application, an instructor is the host to guide students to explore the virtual Reference Construction Site. Figure 5 demonstrates the six core functions in the multiplayer online DT, (1) Server-Client networking system, (2) preparation Lobby, (3) virtual Reference Construction Site, (4) IoT-enabled construction machinery, (5) interactive construction processes, and (6) real-time instruction and communication.

Connecting via Steam Server or VPN, students can search and join different groups created by different instructors. The students first join in the Lobby to meet their instructor and get basic instructions, such as safety regulations and interaction methods. Then they will be led into the virtual site for free exploration. On site, the students can observe various construction processes or choose a type of machine or a construction process to interact with under live instructions. During the tour, students can communicate or ask questions via the chat window. The networking module in UE is able to replicate the dynamic changes of the environment and user status among the clients. Additionally, the implementation of MQTT synchronises the virtual models with the actual object, such as machines and building elements. For example, students can observe the DT of a real robotic fabrication as well as send commands to operate the robot (as shown in Fig. 5, Nr.5).

**Fig. 6:**
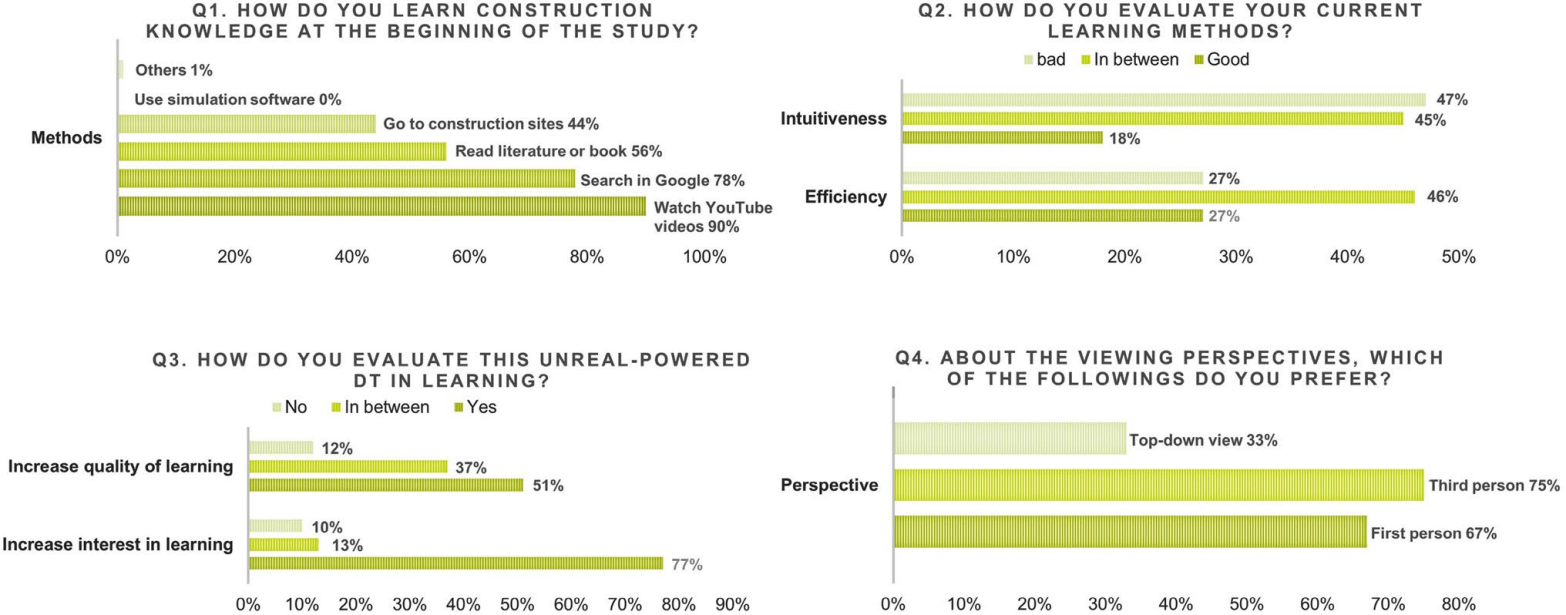
The results of the multiple-choice questions from Q1 to Q4

This use case for education was tested online by the 15 master students in the direction of construction. Feedback from individual student was given to the authors in the form of a questionnaire of 6 questions and an open discussion on Zoom meeting. Figure 6 shows the results of the multiple-choice questions from Q1 to Q4, focusing on the current learning methods of construction and the DT learning experiences. Tables 1 and 2 presents the answers of two open questions Q5 and Q6: the advantages and disadvantages of those methods.

## Result evaluations

### User feedback

This section focuses on the user feedback of the participants in the multiplayer online DT (shown in Fig. 6, Tables 1 and 2). As we can see from the statistics in Q1, for learning construction knowledge, most of the students selected more efficient learning methods of watching videos via YouTube and Google searching than directly reading books or going to construction sites. 90% of the students enjoy learning construction by watching videos, which visually explain the complex processes. Nevertheless, regarding the intuitiveness and efficiency of these learning methods in Q2, only 18% and 27% of the students thought that the current methods have performed well in assisting their study. According to the open discussion, most students saw intuitiveness, which is not sufficient in existing methods, as a key factor and motivation of succeed in knowledge acquisition and comprehension.

Results of Q3 showed that, after experiencing the online DT, 77% of the students thought that the interactive tool increased their interest in learning construction. One student wrote in the questionnaire that, when she saw a real construction site for the first time, she got demotivated by the chaotic environment. The photorealistic game-like DT aroused her interest to learn and explore the construction site. Additionally, 51% students thought the DT increased the quality of learning by observing the real processes in the DT; however, 37% students were still not sure if they can learn better with this application for a deeper understanding.

Considering some users may have 3D sickness after the long-time exposure of three-dimensional graphics, Q4 was concerning about their feelings on different perspectives. Regarding the balance between comfort and immersive experience, some students suggested having the freedom to change perspectives according to different scenarios.

In the open discussion of Q5, many students stated that the construction site is the best place for learning, because one can observe the most authentic processes and discover real problems. However, as students, visits to construction sites were unfortunately not always available for them, especially during COVID19. To conclude, students hoped to learn the most cutting-edge construction knowledge via a more efficient and intuitive approach.

In the answers of Q6, the students expressed their appreciation for the synchronicity between the Reference Construction Site and the learning tool. They also enjoyed getting instant help remotely via the live chat and user interface. Nevertheless, one of the drawbacks mentioned by the students was the large download file of the DT (3.26 GB) before execution. A light-weight method was wished for the next version. In addition, some students thought that the learning tutorials were not systematically categorized. The free exploration seemed more suitable to first semester students for a general impression on construction, rather than a tool for in-depth learning.

**Table 1:** Q5: What are the advantages and disadvantages of your current learning methods?

Advantages	Disadvantages
1. Watch YouTube videos	
- Intuitive and interesting	- Not interactive with fixed viewing angle
- Visualized details of complex processes	- Sometimes not showing the reality
2. Search in Google	
- Rich and detailed information source	- Not interactive with fixed viewing angle
- Very flexible, can do it at anytime and anywhere	- Time-consuming to find the right answers
3. Read literature or books	
- Detailed, scientific and professional description	- Language gap between mother tongue and English
- Provide academic and systematic learning methods	- Sometimes provide obsolete or theoretical information
4. Go to construction sites	
- Best places to learn about construction processes	- Not always available, chaotic and unsafe on-site
- Show the reality, practical real-time learning	- Not easy to observe different parts of the site
5. Use 3D simulation software	
- Very intuitive and interactive	- Cannot find the right one for construction

**Table 2:** Q6: What do you think are the advantages and disadvantages of this DT learning tool according to your experience?

Advantages	Disadvantages
- Reflect the reality on the construction sites	- Large file to download before running the application
- Intuitive in a dynamic virtual environment	- High requirements on the graphic card of a local machine that runs the DT
- Play and learn via real-time interaction	- Misleading introduction might cause serious problems in actual operation
- Remote participation through networking	- Tutorials should be categorized into different machinery and processes beside the free construction exploration
- Flexibility in where, when and how to use	- Not covering all the aspects on construction sites
- Live communication and instruction	- Cannot replace the experience at real construction sites
- Quick and general understanding of various and complex processes	- The current application is more suitable for first semester students

**Fig. 7:**
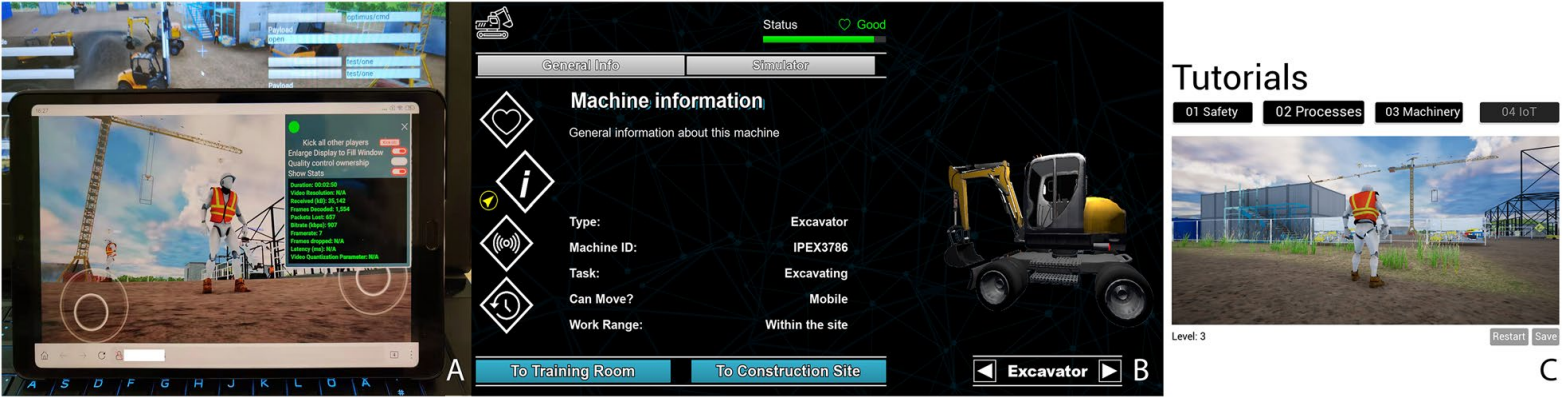
Improvements made according to the students’ feedback

To improve the DT, the integration of user feedback was considered and made after the interview:

- Implementation of the web-browser-based Pixel Streaming for a light-weight application (Fig. 7A)
- Categorization of tutorials and introduction of an achievement system (Fig. 7B and C)
- Connection to an industrial simulator for in-depth applications (see Sect. 4.2)

The next phase of development will integrate more scientific methods (such as game psychology, machine learning, artificial intelligence) to enhance the education function in the DT.

### Bidirectional communication

As mentioned previously, the bidirectional communication was realized via MQTT. This was enabled in the DT by writing a UE-plugin using a MQTT C library^1^. As an application scenario, a KUKA robot was replicated in the DT using a cloud remote control backend - KUKA|crc by Robots in Architecture Research^2^, which calculates kinematics data at its cloud server (Fig. 6). The cloud server receives commands via MQTT and returns the calculated data to the user as well as the robot using the same protocol. In this paper, KUKA|crc served as the backend calculator for the DT, which visualized and twinned the robot status. The result from the research shows that the DT performed well in synchronisation using MQTT, with an interval time for data update between 0.25s and 0.5s. Higher update frequency can cause the DT to freeze. Interpolation of the virtual robot movement between the messages must be implemented, otherwise some key commands and important intermediate states might be lost. However, the interpolation system for this study is still under optimization.

**Fig. 8:**
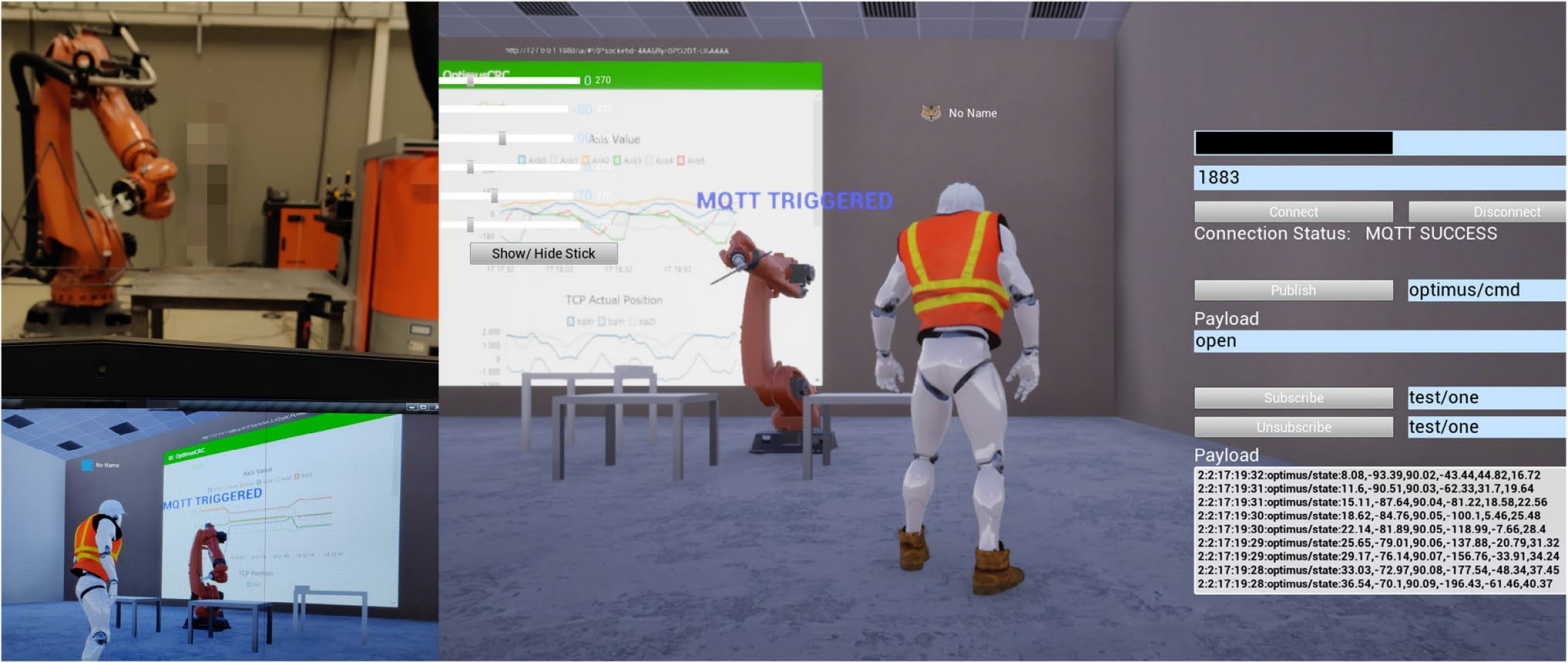
Robotic DT with live-time axis value and tool position plotting

Another useful finding in the research was the web browser function in UE, which allows user to explore websites in the application. This enabled real-time data plotting inside the DT by simply browsing to an online data dashboard (Fig. 8). In this case, important real-time data, such as machine axis value, position deviation and abnormal status, can be monitored and controlled on-site via augmented-reality glasses, online dashboard or smart phone.

## Conclusion and outlook

Based on the Reference Construction Site, this research proposed a first development framework of a UE-powered DT supporting the education in construction. The architecture of UE provided a collaborative foundation for the interdisciplinary framework. Derived from the development framework, the multiplayer online DT presented a use case that enhanced the quality and increased the efficiency of the online education. Using MQTT to communicate with the industrial machinery such as robots, the multiplayer online DT reflected real processes for students to learn from. The first implementation and proof of concept received positive feedback. This can be seen as a good indicator on the impact of a DT-based approach in construction education. Furthermore, the future research will focus on the improvement of the in-depth DT-behaviour simulation, and a more comprehensive evaluation of learning impact.

The potentials of this DT are not only in digital education, with the characteristics of interactivity, authenticity, real-time, multi-dimensional model visualization, but the DT is also capable to expand its functions to safety training in construction companies, on-site monitoring in construction management, and control of automation systems.

## Footnotes

1 paho.mqtt.c https://github.com/eclipse/paho.mqtt.c

2 KUKA|crc https://www.robotsinarchitecture.com/crc/
